# Prevalence and correlates of non-fatal overdose among people who use drugs: findings from rapid assessments in Massachusetts, 2017–2019

**DOI:** 10.1186/s12954-021-00538-9

**Published:** 2021-08-30

**Authors:** Shikhar Shrestha, Thomas J. Stopka, Jaclyn M. W. Hughto, Patricia Case, Wilson R. Palacios, Brittni Reilly, Traci C. Green

**Affiliations:** 1 Department of Public Health and Community Medicine, Tufts University School of Medicine, Boston, MA USA; 2 Departments of Behavioral and Social Sciences and Epidemiology, Brown University School of Public Health, Center for Health Promotion and Health Equity, Brown University, Providence, RI USA; 3 Bouvé College of Health Sciences, Northeastern University, Boston, MA USA; 4 School of Criminology and Justice Studies, University of Massachusetts, Lowell, MA USA; 5 Massachusetts Department of Public Health, Bureau of Substance Addiction Services, Boston, MA USA; 6 Opioid Policy Research Collaborative, Heller School for Social Policy and Management, Brandeis University, Waltham, MA USA

**Keywords:** Non-fatal opioid overdose, People who use drugs, Rapid assessment, Massachusetts, Fentanyl

## Abstract

**Background:**

People who experience non-fatal overdose (NFOD) are at high risk of subsequent overdose. With unprecedented increases in fentanyl in the US drug supply, many Massachusetts (MA) communities have seen a surge in opioid-related overdoses. The objective of this study was to determine factors associated with lifetime and past year NFOD in at-risk MA communities.

**Methods:**

We conducted multiple rapid assessments among people who use drugs (PWUD) in eight MA communities using non-probability sampling (purposive, chain referral, respondent-driven) methods. We collected sociodemographic, substance use, overdose history, substance use treatment, and harm reduction services utilization data. We examined the prevalence of NFOD (lifetime and past year) and identified factors associated with NFOD through multivariable logistic regression analyses in a subset of 469 study participants between 2017 and 2019.

**Results:**

The prevalence of lifetime and last year non-fatal opioid overdose was 62.5% and 36.9%, respectively. Many of the study participants reported heroin (64%) and fentanyl (45%) use during the 30 days preceding the survey. Nonprescription buprenorphine and fentanyl use were independently associated with higher odds of lifetime NFOD, while marijuana use was associated with lower odds of lifetime NFOD (*p* < 0.05). Injection as the route of administration, benzodiazepine, nonprescription buprenorphine, heroin, and fentanyl use were independently associated with higher odds, while methadone use was associated with lower odds of past year NFOD (*p* < 0.05).

**Conclusion:**

We documented a high prevalence of past year and lifetime NFOD among PWUD in MA. Our findings provide indicators that can help inform interventions to prevent overdoses among PWUD, including overdose prevention, medication treatment, and naloxone distribution.

## Introduction

In the USA, the opioid overdose epidemic has exacted an immense cost of human life, with over 400,000 lives lost over the last two decades [1]. The epidemic has also had a significant economic impact, with estimated costs totaling $631 billion between 2015 and 2018 alone [2]. Increase in the prescription of prescription opioids and subsequent increase in opioid overdose deaths marked the first wave of the opioid overdose epidemic [3, 4]. It was followed by second wave of the opioid overdose epidemic, characterized by the rise in heroin use and heroin-related overdose deaths in the early to mid-2010s [4, 5]. The third wave of the opioid overdose epidemic began with the surge in illicit fentanyl-related overdose in recent years [4, 6]. Non-fatal overdoses (NFOD) represent a significant and ever-growing problem among individuals with substance use disorder. Moreover, people who experience NFOD are at a very high risk of subsequent overdose [7–10], and are also at risk of other comorbidities [11, 12]. Furthermore, accurate surveillance of NFOD is a challenge as these events are often only reported when a patient receives documented medical services such as emergency department visits and hospitalization [13, 14]. Understanding the prevalence and factors associated with overdose events is critical to guide future interventions.

In Massachusetts (MA), opioid-related fatal overdoses have steeply increased over the past 20 years, from 375 in 2000 to an estimated 2,104 in 2020. In 2019, fentanyl was detected in toxicology screens in 92% of fatal opioid overdoses [15]. Expanded access to and utilization of the overdose reversal drug, naloxone (brand name Narcan), is necessary to prevent fatal overdoses and research suggests that the rates of NFOD have also been climbing, with the number of emergency medical services (EMS)-administered naloxone rescues reaching approximately 15,000 in 2019 [16]. The risk of one-year mortality in people who experienced NFOD and received treatment at an emergency department is approximately 5% in MA [17]. While positive changes in state health policy have been introduced in recent years, including a 7-day limit to first opioid analgesic prescription, implementation of a prescription drug monitoring program, initiation of new syringe services programs, and development of statewide overdose education and naloxone distribution program, high rates of opioid-related overdoses have persisted in MA and present a substantial public health challenge, meriting further research. In the recent years, the success of these safe injection sites in other nations [18, 19] has prompted increased interest in establishment of safe injection sites in the USA. A recent letter to the editor published in NEJM describes the effectiveness of an unsanctioned injection site in the USA [20, 21], and in July 2021, Rhode Island passed legislation to plan and pilot safe consumption spaces.

Existing studies examining factors associated with opioid use and overdose indicate that young adults, men, non-Hispanic (NH) white people, individuals with less than a high school education, and homeless people are at increased risk of illicit opioid use and adverse opioid-related health outcomes, including overdose [4, 22–25]. However, detailed assessment substance use patterns and experiences in people who use drug is necessary given the high rates of opioid-related overdoses in MA. To that end, the goal of the current study was to estimate the prevalence of NFOD and determine factors associated with lifetime and past year NFOD in high-risk MA communities to better understand which groups may be at greatest risk for NFOD as well as to inform targeted interventions to combat the opioid overdose epidemic.

## Methods

### Study population and recruitment

Between August 2017 and November 2019, we conducted mixed-methods rapid assessments with people who use drugs (PWUD) in MA [26, 27]. Individuals were eligible for the study if they were 18 years of age or older, a resident of MA, and reported using an illicit drug in the past 30 days. Individuals who used marijuana alone were not eligible, as marijuana has been legal in the state of MA since 2016. In this paper, we present the findings from the quantitative data collected during the study.

We selected geographic regions of MA in which surveillance data showed a rising trend in fatal overdoses between 2016 and 2017 [28]. These regions included: Lowell, Lawrence, Quincy, Upper Cape Cod (Barnstable, Mashpee, Yarmouth, Falmouth), Springfield, Chicopee, the North Shore (Lynn, Salem, Beverly, Peabody), and New Bedford. In preparation for recruitment, we conducted environmental scans comprised of a review of publicly available public health and surveillance data, community walk-throughs, and meetings with community partners to identify locations for participant recruitment. We partnered with local organizations (e.g., syringe services programs (SSPs), homeless shelters, health centers) to facilitate the recruitment of potential participants. The detailed methodology used for the recruitment of study participants is described elsewhere [29]. The study design was adapted from World Health Organization Rapid Assessment and Response guide [30]. Briefly, we used a combination of non-probability sampling methods (primarily purposive, chain referral, and respondent-driven) to recruit participants. Potential participants were screened for eligibility by phone or in person. We obtained verbal informed consent from all participants before initiating study procedures. All participants completed a one-time, interviewer-administered survey on an electronic tablet or on paper. The survey assessed sociodemographic variables, information on substance use in the last 30 days, route of substance use, history of overdose experiences and overdose response, and knowledge and experiences about substance use treatment and harm reduction services. The survey was administered in English and Spanish and took approximately 45 min. Each participant received a $20 gift card upon completion of the survey and up to $15 ($5 each) for referring up to three other eligible participants. The study was approved by the Institutional Review Boards of the Boston University Medical Campus and the Massachusetts Department of Public Health.

### Study measures

1. Outcome variables (non-fatal overdose)

We obtained data to assess lifetime and past year experiences with NFOD by first asking study participants whether they had ever experienced a drug-related overdose. If participants answered in the affirmative, we asked for the month and year when they experienced their most recent overdose. We then calculated the time between the survey date and the date of the participant's last overdose to determine whether the NFOD had occurred during the past year. For this study, we used lifetime NFOD and last year NFOD as outcome measures. We also collected information on the details regarding the most recent NFOD such as substance used during the most recent NFOD and whether the participant was taken to a hospital after the overdose.

2. Sociodemographic variables

We operationalized age into three categories: less than 30 years (young adults), 30–45 years, and more than 45 years of age based on the data distribution. We combined race and ethnicity into four independent categories: NH white, Hispanic, NH Black, and NH other. We categorized educational attainment into two groups: less than high school and high school graduate or higher. We also evaluated other sociodemographic indicators: housing status (binary: housed vs. unhoused), unemployment status (binary: yes/no), insurance type (categorized as public vs. other), history of arrest (yes/no), history of incarceration (binary: yes/no), and involvement in the sex trade (binary: yes/no).

3. Substance use variables

We examined substance use in the last 30 days, including use of crack, cocaine, methadone (prescribed), buprenorphine (obtained through one's own prescription), buprenorphine (not obtained by one's own prescription), amphetamine, benzodiazepine, pain medication (which included non-opioid pain medications), fentanyl, heroin, and marijuana. Based on the route of administration of the substances, we also created four dummy variables that indicated how these substances were taken: orally, snorted, smoked, and injected. The dummy variables were non-exclusive (i.e., an individual could have used substances by several routes) and non-specific (i.e., the route of administration was not specific to a type of drug or substance).

4. Naloxone knowledge and access

We asked the study participants whether they knew about naloxone (yes/no). Based on their response to the naloxone knowledge questions (i.e., if answered "yes"), the study participants were also asked whether they currently had naloxone on them (yes/no), had been trained for naloxone administration (yes/no), knew where to get naloxone (yes/no), and to rate the difficulty of obtaining naloxone (categorized into binary variables: easy/extremely easy vs. neutral/difficult/extremely difficult). In our analysis, for individuals who did not know about naloxone, we imputed the values for naloxone branching logic questions with a missing response as "no."

### Data analysis

We excluded participants who did not have a history of opioid use to reduce the risk of combining opioid overdoses with stimulant overdose. We calculated descriptive statistics for all study variables of interest. Using Chi-squared and Fisher exact tests, we examined global differences in sociodemographic variables, substance use, and naloxone knowledge items by the history of NFOD (ever and last year). We used logistic regression models to identify sociodemographic, substance use, and harm reduction knowledge factors that were associated with past year and lifetime NFOD. Through bivariate analyses, we identified covariates that were significant at *p* < 0.20 for consideration in multivariable models. We assessed potential multicollinearity between covariates by estimating the variance inflation factor (VIF); variables with a VIF of 10 or more were removed from the final models. We finally eliminated duplicative variables and those not conceptually distinct. We also used site random effects to account for the differences in recruitment sites, recruitment methods, and recruitment time. We conducted all analyses in SAS 9.4 (Cary, North Carolina).

## Results

We recruited 494 PWUDs between 2017 and 2019. After excluding non-opioid users, we retained 469 participants. Most study participants were male (61.9%), NH white (59.7%), and between the ages of 30–45 years (50.8%). More than one in four participants (28.4%) did not complete high school, and 60.0% were unemployed. Approximately 88.3% of the participants had a history of past arrest and 62.2% had a history of incarceration. The prevalence of lifetime NFOD was 62.5%, and 36.9% of the sample reported experiencing NFOD within one year of the survey (Table 1). Sixty-four percent of the participants reported using heroin, and 45% reported using fentanyl in the last 30 days. Participants also commonly reported the use of buprenorphine (nonprescription; 10.7%) and marijuana (43.1%). Only 15% of the participants reported using pain medications. The most frequently mentioned route of administration was injection (59.5%). More than eight in ten participants reported ever receiving formal drug treatment. Additionally, 97.2% reported that they knew about naloxone. A substantial proportion (62.7%) had naloxone with them at the time of the survey, and 89.8% knew where to get naloxone in the community.

**Table 1:** Sociodemographic characteristics, substance use patterns, and knowledge of naloxone in RACK study participants, Massachusetts, 2017–2019 (*n* = 469)

Variables	Category	Total responses (*N*)	Percentage who had any overdose % (*n*)	*p*-value (2 sided)	Percentage who had last year overdose % (*n*)	*p*-value (2 sided)
Gender	Female	177	38.3 (111)	0.904	38.6 (66)	0.857
	Male	288	61.7 (179)		61.5 4 (105)	
Race	NH^a^ white	280	64.0 (188)	0.049	61.3 (106)	0.85
	Hispanic	125	23.8 (70)		24.9 (43)	
	NH Black	35	5.8 (17)		6.9 (12)	
	NH other	29	6.5 (19)		6.9 (12)	
Age	< 30 years	117	23.8 (70)	0.588	21.4 (37)	0.369
	30–45 years	238	50.7 (149)		52.6 (91)	
	> 45 years	113	25.5 (75)		26.0 (45)	
Education	High School or more	335	71.7 (210)	0.955	69.4 (120)	0.416
	Less than HS	133	28.3 (83)		30.6 (53)	
Employment status	Unemployed	281	59.2 (174)	0.622	58.4 (101)	0.574
	Employed	187	40.8 (120)		41.6 (72)	
Housing status	Housed	151	33.3 (98)	0.521	36.4 (63)	0.141
	Not Housed	317	66.7 (196)		63.6 (110)	
Insurance type	Other	28	4.4 (13)	0.059	4.1 (7)	0.166
	Public	436	96.6 (280)		95.9 (166)	
Traded sex for money*	Yes	118	38.7 (82)	0.027	39.7 (50)	0.104
	No	227	61.3 (130)		60.3 (76)	
History of incarceration	Yes	291	69.6 (204)	< 0.001	68.0 (117)	0.047
	No	177	30.4 (87)		32.0 (45)	
*Substance use in the last 30 days*						
Crack	Yes	276	64.9 (179)	0.246	39.5 (109)	0.162
Cocaine	Yes	303	63.0 (191)	0.832	39.3 (119)	0.148
Methadone	Yes	109	67.9 (74)	0.2	30.3 (33)	0.103
Buprenorphine (Rx^b^)	Yes	128	71.1 (91)	0.021	43.0 (55)	0.094
Buprenorphine (non-Rx)	Yes	50	76.0 (38)	0.04	52.0 (26)	0.019
Amphetamine	Yes	38	76.3 (29)	0.07	47.4 (18)	0.162
Benzodiazepine	Yes	127	72.4 (92)	0.008	44.9 (57)	0.162
Fentanyl	Yes	211	76.3 (161)	< 0.001	47.4 (100)	< 0.001
Heroin	Yes	301	69.8 (210)	< 0.001	44.9 (135)	< 0.001
Marijuana	Yes	202	55.9 (113)	0.009	33.2 (67)	0.1466
Pain medication	Yes	71	13.6 (294)	0.230	24 (13.9)	0.599
*Route of administration of substance use*						
Oral	Yes	150	66.7 (100)	0.223	46 (69)	0.005
Smoke	Yes	280	63.2 (177)	0.774	39.3 (110)	0.19
Snort	Yes	208	54.8 (114)	0.002	32.7 (68)	0.093
Inject	Yes	279	77.8 (217)	< 0.001	47.3 (132)	< 0.001
Snort or inject	Yes	407	66.1 (269)	< 0.001	39.6 (161)	0.002
*Naloxone knowledge*						
Do you know what naloxone is?	Yes	456	63.2 (288)	0.211	37.3 (170)	0.295
Do you currently have naloxone?	Yes	294	68.4 (201)	0.001	39.8 (117)	0.091
Have you ever been trained to use naloxone?	Yes	350	66.6 (233)	0.003	38.3 (134)	0.282
Do you know where to get Narcan?	Yes	421	65.3 (275)	0.001	38.0 (160)	0.137
How easy/difficult is it to get Narcan?	Easy/extremely easy	360	66.7 (240)	0.047	38.1 (137)	0.445
	Neutral/difficult/extremely difficult	65	53.8 (35)		43.1 (28)	

^*^Variable not assessed in first site (Lowell) hence not included in multivariable analysis

^a^*NH* non-Hispanic

^b^*Rx* prescription

At the time of the most recent NFOD (in the last year), a majority of the participants reported using heroin (120/170) or fentanyl (97/170 [information not shown in table]). Polysubstance use at last NFOD was common, with 109 participants reporting having used two or more drugs during the overdose episode. Ninety-four participants reported that they were transported to the hospital for their most recent NFOD, and of those, only 37.2% rated their experience there as positive (vs. 26.6% neutral, 36.2% negative).

In our bivariate analyses, drug use and harm reduction service use were significantly associated with lifetime and past year history of NFOD (Table 2). The use of prescription buprenorphine, nonprescription buprenorphine, benzodiazepines, fentanyl, and heroin was all significantly and positively associated with history of lifetime NFOD (*p* < 0.05). Marijuana use was negatively associated with history of lifetime NFOD (*p* < 0.05). The use of nonprescription buprenorphine, benzodiazepines, fentanyl, and heroin was significantly associated with last year NFOD (*p* < 0.05). Injection as the route of administration was significantly associated with increased odds of both any and last year NFODs, whereas snorting was associated with decreased odds of any NFOD (both last year and lifetime), and oral substance use was associated with decreased odds of last year fatal overdose (*p* < 0.05). Additionally, we observed that having naloxone on hand, being trained for naloxone use, knowing where to get naloxone, and perceived difficulty in getting naloxone in the community were all significantly associated with higher odds of having a history of lifetime NFOD (*p* < 0.05).

**Table 2:** Bivariate factors associated with non-fatal overdose (ever, last year) among RACK study participants, Massachusetts, 2017–2019 (*n* = 469)

		Ever experienced non-fatal opioid overdose		Last year non-fatal opioid overdose	
Variables	Comparison	OR^a^ (95% CI^b^)	*p*-value	OR (95% CI)	*p*-value
Gender	Female vs male	1.02 (0.70–1.51)	0.904	1.04 (0.70–1.53)	0.857
Race	Hispanic vs NH^c^ other	0.67 (0.29–1.56)	0.448	0.74 (0.33–1.7)	0.569
	NH black vs NH other	0.50 (0.18–1.37)	0.111	0.74 (0.27–2.04)	0.689
	NH white vs NH other	1.08 (0.48–2.41)	0.048	0.86 (0.40–1.88)	0.813
Age	30–45 years vs > 45 years	0.85 (0.53–1.36)	0.588	0.94 (0.59–1.48)	0.371
	Less than 30 years vs > 45 years	0.76 (0.44–1.29)		0.70 (0.41–1.20)	
Education	High school or more vs less than high school	1.01 (0.67–1.53)	0.955	0.84 (0.56–1.27)	0.416
Employment	Employed vs unemployed	1.10 (0.75–1.62)	0.622	1.12 (0.76–1.64)	0.574
Housing	Housed vs not housed	1.14 (0.76–1.71)	0.521	1.35 (0.91–2.01)	0.142
Insurance	Public vs other	2.07 (0.96–4.46)	0.063	1.84 (0.77–4.43)	0.172
Traded sex for money*	Yes vs no	1.70 (1.06–2.72)	0.028	1.46 (0.93–2.31)	0.104
*Substance use in the last 30 days*					
Crack	Yes vs no	1.25 (0.86–1.83)	0.246	1.32 (0.90–1.93)	0.162
Cocaine	Yes vs no	1.04 (0.71–1.54)	0.832	1.34 (0.90–2.00)	0.148
Methadone	Yes vs no	1.35 (0.85–2.12)	0.201	0.68 (0.43–1.08)	0.104
Buprenorphine (Rx^d^)	Yes vs no	1.67 (1.08–2.59)	0.022	1.42 (0.94–2.16)	0.095
Buprenorphine (non-Rx)	Yes vs no	2.02 (1.02–3.97)	0.043	2.00 (1.11–3.62)	0.021
Amphetamines	Yes vs no	2.02 (0.93–4.37)	0.075	1.60 (0.82–3.12)	0.165
Benzodiazepine	Yes vs no	1.82 (1.17–2.84)	0.008	1.59 (1.05–2.40)	0.029
Fentanyl	Yes vs no	3.03 (2.03–4.52)	< 0.001	2.28 (1.56–3.35)	< 0.001
Heroin	Yes vs no	2.31 (1.56–3.41)	< 0.001	2.78 (1.82–4.26)	< 0.001
Marijuana	Yes vs no	0.60 (0.41–0.88)	0.009	0.75 (0.51–1.10)	0.147
Pain medication	Yes vs no	0.73 (0.44–1.22)	0.231	0.85 (0.50–1.45)	0.559
*Route of substance use*					
Oral	Yes vs no	1.29 (0.86–1.94)	0.222	1.76 (1.18–2.62)	0.005
Smoke	Yes vs no	1.06 (0.72–1.55)	0.773	1.29 (0.88–1.90)	0.190
Snort	Yes vs no	0.55 (0.37–0.80)	0.002	0.72 (0.49–1.06)	0.093
Inject	Yes vs no	5.14 (3.43–7.70)	< 0.001	3.26 (2.15–4.96)	< 0.001
Snort or inject	Yes vs no	2.88 (1.67–4.99)	< 0.001	2.73 (1.41–5.28)	0.003
*Naloxone knowledge and access*					
Do you *know what naloxone/Narcan* is?	Yes vs no	2.00 (0.66–6.05)	0.22	1.98 (0.54–7.3)	0.304
Do you currently *have naloxone/Narcan* kit with you?	Yes vs no	1.91 (1.30–2.80)	0.001	1.41 (0.95–2.08)	0.091
Have you ever been *trained to use naloxone/Narcan*?	Yes vs no	1.89 (1.24–2.89)	0.003	1.27 (0.82–1.97)	0.282
Do you know *where to get naloxone/Narcan*?	Yes vs no	2.87 (1.56–5.30)	0.001	1.65 (0.85–3.21)	0.141
Around here, *how easy would you say it is to get naloxone/Narcan* to take home with you?	Easy/extremely easy vs neutral/difficult/extremely difficult	0.58 (0.34–1.00)	0.048	1.23 (0.72–2.10)	0.445

^*^Variable not assessed in first site (Lowell) hence not included in multivariable analyses

^a^OR: odds ratio

^b^*CI* confidence interval

^c^*NH* non-Hispanic

^d^*Rx* prescription

In the multivariable models, after adjusting for sociodemographic variables, random effects of site, and other factors, we found that nonprescription buprenorphine use (adjusted odds ratio [aOR]: 1.9, 95% [confidence interval] CI: 1.2–3.0) and fentanyl use (aOR: 2.4, 95% CI: 1.6–3.8) were significantly and independently associated with higher odds of a history lifetime NFOD, while marijuana use (aOR: 0.6, 95% CI: 0.3–0.98) was negatively and independently associated with a NFOD history (Table 3). Benzodiazepine (aOR: 1.6, 95% CI: 1.1–2.4), nonprescription buprenorphine (aOR: 2.1, 95% CI: 1.2–3.6), heroin (aOR: 1.9, 95% CI: 1.1–3.5), and fentanyl (aOR: 1.6, 95% CI: 1.1–2.2) use along with injection as the route of administration (aOR: 2.6, 95% CI: 1.6–4.4) were all positively associated with last year NFOD, holding all else constant. Only current methadone use (aOR: 0.4, 95% CI: 0.3–0.6) was significantly and independently associated with lower odds of past year NFOD (Table 4). Among the sociodemographic variables considered when compared to NH Whites, only Hispanic (aOR: 0.6, 95% CI: 0.4–0.99) and NH Black race (aOR: 0.6, 95% CI: 0.4–0.9) were associated with lower odds of lifetime NFOD. For the past year NFOD experience, only people who identified as Hispanic (vs. NH White people) were at lower risk (aOR: 0.5, 95% CI: 0.3–0.9). The odds of experiencing past year non-fatal overdose for people who use drugs identifying as NH Black were not significantly different from people who use drugs who identified as NH White.

**Table 3:** Factors independently associated with ever overdose among RACK study participants, Massachusetts, 2017–2019

Variable	Comparison	aOR^a^	95% CI^b^	*p*-value
Gender	Female vs male (ref)	0.82	0.51–1.31	0.400
Race	Hispanic vs NH^c^ white (ref)	0.63	0.4–0.99	0.045
	NH other vs NH white (ref)	1.11	0.42–2.92	0.828
	NH black vs NH white (ref)	0.60	0.40–0.91	0.016
Age	Less than 30 years vs more than 45 years (ref)	0.64	0.40–1.00	0.051
	30–45 years vs more than 45 years (ref)	0.74	0.49–1.13	0.165
Education	High school or more vs less than HS (ref)	0.94	0.62–1.45	0.793
*Have naloxone/Narcan*	Yes vs no (ref)	1.49	0.85–2.60	0.168
Drug use route: snort or inject	Yes vs no (ref)	1.72	0.98–3.02	0.058
*Substance use in the last 30-days*				
Alcohol	Yes vs no (ref)	0.94	0.62–1.43	0.781
Rx^d^ Buprenorphine	Yes vs no (ref)	1.36	0.91–2.04	0.129
Non-Rx Buprenorphine	Yes vs no (ref)	1.94	1.24–3.02	0.004
Amphetamine	Yes vs no (ref)	1.42	0.69–2.89	0.338
Benzodiazepine	Yes vs no (ref)	1.49	1.00–2.23	0.052
Fentanyl	Yes vs no (ref)	2.44	1.58–3.77	< .0001
Heroin	Yes vs no (ref)	1.40	0.88–2.21	0.153
Marijuana	Yes vs no (ref)	0.57	0.33–0.98	0.042

^a^*aOR* adjusted odds ratio

^b^*CI* confidence interval

^c^*NH* non-Hispanic

^d^*Rx* prescription

**Table 4:** Factors independently associated with past year overdose among RACK study participants, Massachusetts, 2017–2019

Variable		aOR^a^	95% CI^b^	*p*-value
Gender	Female vs male (ref)	1.01	0.74–1.37	0.955
Race	Hispanic vs NH^c^ white (ref)	0.54	0.31–0.92	0.024
	NH other vs NH white (ref)	0.79	0.47–1.34	0.378
	NH black vs NH white (ref)	1.07	0.90–1.28	0.460
Age	Less than 30 years vs more than 45 years (ref)	1.31	0.67–2.55	0.435
	30–45 years vs more than 45 years (ref)	1.01	0.48–2.13	0.974
Education	High school or more vs less than HS (ref)	0.88	0.45–1.72	0.701
Housed	Yes vs no (ref)	0.98	0.68–1.41	0.903
*Have naloxone/Narcan*	Yes vs no (ref)	0.93	0.63–1.37	0.710
Drug use route: inject	Yes vs no (ref)	2.63	1.57–4.4	< 0.001
*Substance use in the last 30-days*				
Crack	Yes vs no (ref)	1.17	0.84–1.63	0.356
Cocaine	Yes vs no (ref)	1.06	0.63–1.76	0.83
Methadone	Yes vs no (ref)	0.42	0.30–0.59	< .0001
Rx^d^ Buprenorphine	Yes vs no (ref)	1.13	0.78–1.64	0.515
Non-Rx Buprenorphine	Yes vs no (ref)	2.10	1.23–3.56	0.006
Amphetamine	Yes vs no (ref)	1.18	0.62–2.22	0.619
Benzodiazepine	Yes vs no (ref)	1.64	1.14–2.37	0.008
Fentanyl	Yes vs no (ref)	1.55	1.08–2.22	0.018
Heroin	Yes vs no (ref)	1.91	1.05–3.46	0.033
Marijuana	Yes vs no (ref)	0.73	0.39–1.37	0.323

^a^*aOR* adjusted odds ratio

^b^*CI* confidence interval

^c^*NH* non-Hispanic

^d^*Rx* prescription

## Discussion

We examined the prevalence and correlates of NFOD among PWUD across MA between 2017 and 2019. We observed a high prevalence of lifetime (62.5%) and past year (37%) overdose in the study population. Nonprescription buprenorphine use and fentanyl were significantly, positively, and independently associated with higher odds of NFOD, whereas marijuana and methadone use were associated with lower odds of NFOD.

The estimates of NFOD prevalence observed in our study are significantly higher than recently published estimates of the history of lifetime NFOD in PWUD, which range from 15 to 58% [8, 31–34]. The rise of fentanyl in the illicit drug supply across the USA has resulted in a high prevalence of NFOD and a sharp rise in fatal OD [35–37]. Forty-five percent of our study participants also reported using fentanyl and among those 76% reported any overdose. Our findings are consistent with previous studies in MA, which have linked 75% to 94% of opioid-related overdoses to fentanyl [35, 38]. Findings from our multivariable regression models also strongly support the association between specific substance use (e.g., heroin use, fentanyl) and use patterns (i.e., injection drug use) with NFOD. These findings are consistent with prior research linking overdose risk to heroin and injection drug use [34, 39]. Criminalization of illicit substances can create an environment where people who use drugs opt toward high-risk behavior such as sharing and reusing needles. Previous studies indicate that such practices can also predispose individuals to the risk of transmitting infectious diseases such as HIV and HCV [12, 40, 41]. In light of these risks and the current HIV outbreak in the state, findings from the present study underscore the urgency of efforts to address the health of people using heroin and fentanyl in MA.

Prior research attributes fatal overdose rates to the high prevalence of fentanyl in the drug market [6, 35, 42]. Access to medication for opioid use disorders (MOUD) and harm reduction services—two evidence-based overdose prevention measures [43–45], have been shown to reduce fatal overdoses in many regions of the USA [9, 43, 46]. In examining access to harm reduction services among PWUD in MA, we found that a high proportion of participants reported carrying naloxone (62.7%) compared to estimates (17%-48%) from studies in other locations [34, 37, 47]. This higher prevalence of naloxone access among participants in our sample is likely due to the fact that MA has been at the forefront of public health responses to the opioid overdose epidemic [46, 48, 49]. Since 2006, MA has provided access to health insurance for all residents. Additionally, MA has implemented state-funded harm reduction services such as overdose education and naloxone distribution since 2005, with additional access to naloxone through pharmacies since 2018 [46, 48–50]. The observation that difficulty in accessing naloxone was associated with NFOD experience suggests there is still much room for improvement in community naloxone provision and equity in who can easily access this lifesaving medication. Further, the state's well-established 9-1-1 Good Samaritan Law, which provides limited immunity for drug-related charges when responding to a suspected overdose emergency, may have contributed to increased overdose rescues and thus improved survival in at-risk individuals, thereby leading to a higher prevalence of having experienced NFOD [46, 48, 49, 51, 52]. However, it is important to note that our study did not specifically examine the details surrounding the participants overdose to conclusively estimate the effect of GSL on the number of overdose rescues.

Participants who reported using non-prescribed buprenorphine in the 30 days before the study interview had approximately twofold increased odds of having experienced NFOD during the past year and in their lifetime, whereas current use of prescribed buprenorphine was not associated with NFOD. Buprenorphine is a key medication for the effective treatment of OUD [53]. But like all medication treatments for chronic conditions, it is most effective with prolonged and consistent therapeutic use. It is generally understood that the use of nonprescription buprenorphine is an attempt to self-treat withdrawal symptoms associated with opioid dependence, which is facilitated by buprenorphine's low risk of adverse events, a safety profile as a partial agonist, and greater availability [54–57]. Prior studies have reported a lower risk of NFOD with non-prescribed buprenorphine use [34, 56], though neither were conducted in places where or during periods when fentanyl was the dominant opioid being consumed. As we are unable to establish temporality due to the cross-sectional nature of the study design, it is difficult to disentangle whether the withdrawal symptoms may have played in mediating NFOD risk. It is possible that individuals who have had prior overdoses may be motivated to utilize non-prescribed buprenorphine as a means to reduce their risk of overdose and also serve as the first step to initiation of formal treatment [54–56]. The utilization of nonprescription of buprenorphine represents a complex dynamic of interaction between people with OUD and their substance use behaviors, substance use treatment providers, substance use treatment policy, and rurality. In states where prescribed buprenorphine access is limited due to stricter regulation of MOUD treatment, the presence of higher substance use-related stigma, or rurality, use of nonprescription buprenorphine may confer added protection to an individual with OUD [58]. In MA, where MOUD is more easily accessible, the use of nonprescription buprenorphine could also be indicative of higher risk-taking behavior and or mistrust of substance use treatment providers [59]. The findings highlight the importance of ensuring access to buprenorphine as a harm reduction measure, decriminalization of buprenorphine diversion, and improved availability through pharmacies [60, 61].

We also observed that the odds of lifetime NFOD were lower in Hispanic and NH Black people compared to NH white people. However, a different pattern of past year NFOD emerged, suggesting similarities with respect to risk, which is in congruence with previously reported findings [10, 62]. The opioid overdose epidemic primarily affected NH white people in the early and mid-2000s owing to the disparities in opioid prescribing [4]. However, as the epidemic evolved, a much broader population is being impacted. A recently published study indicated that the greatest increase in opioid-related mortality was seen in NH Black men, indicating the need to provide focused interventions for a minority population that was previously thought to have lower risks [15, 63]. The odds of past year NFOD were no different between NH white and Blacks; thus, our sample corroborates the recent and concerning fatal overdose trends nationally by race and, for the first time in the literature, suggests that the alarming increase appears to extend to NFOD events as well.

The findings from our study also indicate that methadone and marijuana use is associated lower odds of having a history of NFOD, even while using other illicit drugs in the past month. Methadone is commonly used in the treatment of OUD. Given that methadone maintenance reduces opioid cravings and that there is relatively limited diversion risk, the association between methadone and lower odds of past year NFOD suggests that methadone has been effective in reducing behaviors (e.g., fentanyl use, injection of drugs) and circumstances (e.g., incarceration, loss of tolerance) that increase the risk for fatal overdose. More research, however, is needed to understand the motivation for taking methadone among those with and without a history of NFOD (both lifetime and last year). Additionally, we found a strong inverse association between marijuana use and lifetime NFOD (aOR: 0.6). The legalization of medical marijuana has been shown to reduce the use of prescription drugs in Medicare part D and Medicaid enrollees in some studies [64, 65]. Segura et al. showed no effect of marijuana laws on the non-medical use of opioids [66], while other studies have found that marijuana use is related to an increased risk of having OUD [67–69]. Additional studies show the reduced use of illicit opioids in people who used marijuana frequently [70–73]. The association between marijuana and reduced odds of NFOD could be channeled through several pathways. Marijuana could possibly be used to manage underlying pain, stave off drug cravings, or intentionally limit the use of opioids by active users. Recent legal access to marijuana, for recreational or medical purposes, may encourage reduced use of illicit substances by creating safer, more consistent channels to a mind-altering substance. It is also likely that we observed these associations (reduced risk) because marijuana is more frequently being used by people who do not intentionally use opioids and, therefore, would be at low risk of NFOD.

Our findings should be considered in light of several limitations. We focused on high-overdose-risk communities in MA; hence, our results may not be generalizable to other regions of the state. Additionally, the data we collected were based on self-report, which is prone to recall and reporting bias. The lack of substance use testing can also affect the reported rates of heroin and fentanyl use as many participants could have unknowingly used heroin contaminated with fentanyl. The high rate of naloxone possession observed in our study could also have been due to the oversampling of participants from substance use treatment and harm reduction centers. To protect the confidentiality of participants and encourage participation, exact dates of care and health events were not obtained for this rapid assessment. Month and year of events were self-reported by participants, however, and allowed broadly for exploring the order of exposure if not the precise timing of exposure, which may be better achieved in a different study design. Furthermore, we carried out the rapid assessments over an extended period between August 2017 and November 2019, during which time substance use patterns could have changed. Notably, however, we used a random effects model to account for any variability associated with recruitment location, method, and time. The study also lacked an assessment of mental health issues in the population, which could have a significant association with a history of both lifetime and recent NFOD. Future research with this population could explore behavioral health considerations more broadly as well as motivations for using prescribed, non-prescribed, and illicit substances in relation to overdose risk.

## Conclusion

Our study provides strong evidence indicating that NFOD is a significant issue in MA, driven primarily by factors associated with specific drugs—fentanyl, heroin—and their use by injection. Expected and unexpected fentanyl use continues to pose a considerable challenge to public health efforts to save lives, as is evident even in places like MA, where there are high levels of awareness and use of naloxone. Given the high risk of fatal overdose among people with a history of NFOD [7–10], our findings highlight the need for interventions that promote uptake of MOUD and improved access to naloxone across all PWUDs. Ongoing efforts to ensure equity and access to treatment and harm reduction supplies are needed to effectively address the opioid overdose epidemic and reduce the incidence of NFOD and fatal overdose among PWUDs.

## Data Availability

The datasets collected and analyzed for the current study are not publicly available due to privacy concerns but are available from the corresponding author on reasonable request and pending the approval of the state of Massachusetts Department of Public Health.
